# THE ASSOCIATION OF A FRAILTY INDEX AND INCIDENT DELIRIUM IN HOSPITALIZED VETERANS

**DOI:** 10.1093/geroni/igz038.3305

**Published:** 2019-11-08

**Authors:** Andrea Y Sillner, Robert McConeghy, Caroline Madrigal, Deborah J Culley, Rakesh C Arora, James Rudolph

**Affiliations:** 1 The Pennsylvania State University, University Park, Pennsylvania, United States; 2 Veteran's Health Administration, Providence, Rhode Island, United States; 3 U.S. Department of Veterans Affairs, Providence, Rhode Island, United States; 4 Brigham and Women’s Hospital, Boston, Massachusetts, United States; 5 University of Manitoba, Winnipeg, Manitoba, Canada; 6 Providence RI VA, Providence, Rhode Island, United States

## Abstract

Frailty is an accumulation of deficits that helps identify patients who are vulnerable to stressors. Acute illness and hospitalization are stressors that may result in delirium. Delirium is significant in older adults, resulting in increased hospital stays, institutionalization, morbidity, and mortality. This study aimed to determine if a frailty index (FI), calculated on hospital admission, was associated with the development of incident delirium. An FI was built on an accumulation of deficits model which included assessments of cognition, physical function, and medical comorbidities for a cohort of 218 patients admitted to a Veteran Affairs medical facility. The FI was calculated as a proportion of possible deficits (range 0-1; higher scores indicate increased frailty). Delirium was assessed daily by expert clinician interview. Participants were, on average, 71 years (SD=9.53), white (92.7%), and male (91.7%). Participants were grouped using FI ranges as non-frail (FI<0.25; 26%), pre-frail (FI=0.25-0.35; 39%), and frail (FI>0.35; 35%). Incident delirium was more likely to occur in those who were frail (29.3%, p=0.001), compared to those who were pre-frail (20.9%) or non-frail (3.6%). The association of FI and incident delirium remained after adjustment for age, education, and other demographics (pre-frail: adjusted OR=5.64, 95%CI; 1.23, 25.99; frail: adjusted OR=6.80, 95%CI; 1.38, 33.45). Continued data analysis will include an AUC model to demonstrate robustness of the FI. The results from this study support the use of frailty assessments at hospital admission to identify patients at high risk of delirium and in need of additional clinical support and interdisciplinary resources.

